# Genes and Diet in the Prevention of Chronic Diseases in Future Generations

**DOI:** 10.3390/ijms21072633

**Published:** 2020-04-10

**Authors:** Marica Franzago, Daniele Santurbano, Ester Vitacolonna, Liborio Stuppia

**Affiliations:** 1 Department of Medicine and Aging, School of Medicine and Health Sciences, ‘G. d’Annunzio’ University of Chieti-Pescara, 66100 Chieti, Italy; 2 Center for Advanced Studies and Technology (CAST), ‘G. d’Annunzio’ University of Chieti-Pescara, 66100 Chieti, Italy; 3 DISC-Diversified Integrated Sport Clinic, Dubai 00000, UAE; 4 Department of Psychological, Health and Territorial Sciences, School of Medicine and Health Sciences, ‘G. d’Annunzio’ University of Chieti-Pescara, 66100 Chieti, Italy

**Keywords:** nutrigenetics, nutrigenomics, epigenetics, gene-nutrient interaction, transgenerational effect, non-communicable diseases

## Abstract

Nutrition is a modifiable key factor that is able to interact with both the genome and epigenome to influence human health and fertility. In particular, specific genetic variants can influence the response to dietary components and nutrient requirements, and conversely, the diet itself is able to modulate gene expression. In this context and the era of precision medicine, nutrigenetic and nutrigenomic studies offer significant opportunities to improve the prevention of metabolic disturbances, such as Type 2 diabetes, gestational diabetes, hypertension, and cardiovascular diseases, even with transgenerational effects. The present review takes into account the interactions between diet, genes and human health, and provides an overview of the role of nutrigenetics, nutrigenomics and epigenetics in the prevention of non-communicable diseases. Moreover, we focus our attention on the mechanism of intergenerational or transgenerational transmission of the susceptibility to metabolic disturbances, and underline that the reversibility of epigenetic modifications through dietary intervention could counteract perturbations induced by lifestyle and environmental factors.

## 1. Background

In 1848, the German philosopher Ludwig Feuerbach claimed that “man is what he eats”. Indeed, 160 years after this affirmation, we can confirm that diet is a major factor affecting the quality of life in humans. In fact, diet habits, such as high consumption of fats and sugars, alcohol abuse and reduced vegetable and fruits intake are major components of risk for non-communicable diseases (NCDs), which are characterized by raised blood cholesterol and glucose, hypertension and obesity. Therefore, an appropriate nutrition pattern is recommended as a protective factor against the risk of heart disease, cancer, diabetes and other NCDs, which represent the primary cause of morbidity and mortality worldwide [1].

In recent years, much attention has also been devoted to the role played by diet in human reproduction, not only because infertility represents a major health problem in western countries, but also due to emerging research that corroborates the “developmental origins of health and disease (DOHaD)” hypothesis, which underlines the impact of prenatal, or even preconceptional environmental exposures on the long-term health of the offspring [2].

Although a huge amount of media information has been devoted to the description of the best nutritional approaches to improve human health and reproductive fitness, this approach is still based on the “one size fits all” model, which does not take in account the genetically determined interindividual variability in food metabolism.

In this review, we provide an overview on the recent literature data on this topic and of latest research findings in the fields of nutrigenetics, nutrigenomics and epigenetics.

## 2. Dietary Habits and Human Reproduction

The relationship between diet and fertility in males has been demonstrated by several reports that describe the effects of specific food on semen quality (for a review see Ricci et al. [3]). In particular, it has been suggested that fruit and vegetables, vitamins A, C and E, folates, organ meat and fish have a positive effect on semen quality. On the other hand, trans fat, total fat, processed meat, dairy products and soy phytoestrogens are considered to induce a negative effect on spermatogenesis [3].

Other dietary habits, such as intake of caffeine and tea, have also been suggested to influence male reproductive fitness in terms of fertilization rate, pregnancy rate and miscarriage rate [4].

Confirmation of the relationship between diet and reproductive fitness also comes from studies reporting a positive effect of the Mediterranean diet (MedDiet) on the semen quality of male partners of couples attempting fertility treatment, and on in vitro fertilization (IVF) success rates among non-obese women [5,6].

Due to the current relevance of fertility issues it has been suggested that the influence of diet on fertility could be a specific topic for public health nutrition programs, especially because the correlation between diet and other risk factors assumes the shape of a vicious cycle [7]. In fact, dietary risk factors, such as high intake of saturated fat or sugar, are strongly related to obesity, which in turn represents a risk factor for both male and female infertility. Obesity can be related to psychosocial risk factors, such as depression anxiety and stress, which are well-known to reduce fertility. Furthermore, sociodemographic risk factors could be a possible cause of unhealthy diet. In this view, diet appears to play a central role in the network of environmental factors that are able to affect human health and fertility.

Despite all the data that support a crucial role played by the diet in a couple’s reproductive fitness, some discrepancies can be noted among different studies. Inconsistencies in the reported effect of different foods were analyzed by Gaskins and Chavarro [8], who demonstrated that the role played by some elements in human fertility, such as vitamin D, antioxidants, long chain omega 3, fatty acid and dairy products is not yet clear. Moreover, very recent studies on folic acid and zinc supplementation in male partners did not show an improvement in semen quality or live birth rates in couples undergoing infertility treatment [9]. A possible explanation for these discrepancies is that people are not genetically identical and the presence of specific genetic variants can influence metabolism, therefore, the effect of diet may be variable. In other words, a specific nutrient may provide benefit to some individuals but not to others, based on their different genotype. This makes it necessary to develop personalized nutrition plans based on the different genetic make-up of each subject, that is, nutrigenetic programs [10].

## 3. Nutrigenetics and Human Health

**Figure 1:**
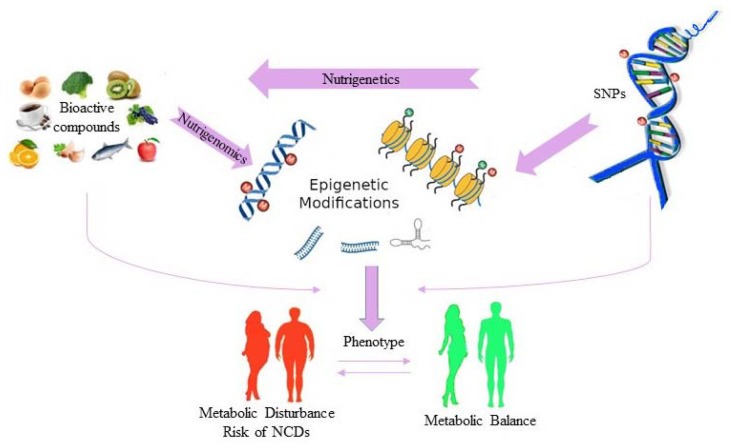
Interactions among genes, diet and human health.

Nutrigenetics can be defined as the field of nutritional genomics, which studies (i) the role of specific genetic variants, in the form of single nucleotide polymorphisms (SNPs), in the modulation of the response to dietary components, and (ii) the implications of such interaction, including the influence on health status and predisposition to nutrition-related diseases [11,12] (Figure 1). Thus, the primary aim of nutrigenetics is to design effective, personalized nutritional strategies that not only result in body weight loss but also prevent metabolic disturbances such as Type 2 diabetes (T2DM), hypertension, dyslipidaemias, and cardiovascular disease (CVD).

Several genes within our genome are known to influence the metabolism of nutrients [13]. The main genes, whose variants are related to body weight loss in response to hypocaloric diets and/or physical activity programs in adults, are involved in the regulation of lipid metabolism and adipogenesis; others are related to carbohydrate metabolism, energy intake and expenditure, and the circadian system [13]. Of note, it has also been demonstrated that variants in taste, olfactory and texture-related genes can influence perception and preferences for certain foods, which affects the susceptibility to nutrition-related conditions [14].

There are well-documented examples of clinically significant nutrigenetic interactions including: (i) saturated fats intake, *APOA2 2265T* > *C* variant and BMI [15]; (ii) coffee intake, gene variants mostly involving adrenergic receptors and hypertensive response [16]; and (iii) folic acid supplementation, *MTHFR* gene variants, homocysteine levels and CVD risk [15].

Among genetic variants related to nutrition, a key role is played by SNPs in the *FTO* gene affecting body weight and body composition. In fact, carriers of the *FTO* rs9939609 *AA* genotype are likely to be more obese than non-carriers of the *A* risk allele [17]. This variant is considered one of the strongest risk factors for polygenetic obesity. Nevertheless, it has been demonstrated that the increased susceptibility to obesity induced by the *A* risk allele can be modified by either physical activity or reduction in energy intake. This provides an example of how the genetic susceptibility to several NCDs can be modulated through positive lifestyle changes [18]. Another study found a gene–diet interaction with the MedDiet for both the *FTO* rs9939609 and for the *MC4R* rs17782313, which showed a higher T2DM risk in carriers of the variant alleles as compared to wild-type subjects when the MedDiet was not complied with. These associations disappear when there is high adherence to the MedDiet [19]. Several studies have also reported the interactions between *TCF7L2* rs7903146 (*C* > *T*) and dietary components in modulating T2DM risk [20,21,22,23]. In fact, wholegrain intake was inversely associated with T2DM risk among *CC* carriers, whereas this protective effect was inhibited by the presence of the *T*-allele. These examples demonstrate the complexity of nutrigenetics in terms of influences, that is, the different genetic predisposition in various populations, as well as the environmental factors that can influence the gene–nutrient association.

Accordingly, based on the specific genotype, different individuals metabolize lipids, carbohydrates and folates in different ways and have a specific response to identical diets. Nutrigenetic tests are currently used in specific circumstances for selecting an appropriate diet in patients at risk of different conditions. For example, our group, by using a simple panel of nine nutrigenetic variants, demonstrated an increased risk of gestational diabetes mellitus (GDM) in women carriers of the *TT* genotype of the *TCF7L2* gene (OR 2.5) [24,25]. Moreover, an association between variants in *PPARG2*, *APOA5*, *MC4R*, *LDLR* and *FTO* genes and lipid parameters has been detected [24,25,26]. The diagnosis of GDM allows the identification of a population that is highly vulnerability to T2DM and metabolic syndrome, providing an easy and ideal tool that matches routine anthropometric and biochemical factors, dietary assessments and genetic make-up in clinical practice [25,26]. The integration of precision nutrition into routine clinical care is a growing challenge, and this approach, once validated, could help improve not only the personalized nutrient intake but also the early identification and stratification of women at increased risk. However, this is just an example of a specific condition in which the daily intake of food and the effect on body size and on clinical parameters can be easily assessed. Moreover, adherence to a specific diet by women affected by a condition that could influence the health of the fetus is expected to be very high. On the other hand, correct information about dietary habits are more difficult to obtain when dealing with the general population and couples trying to have a child.

Some studies have shown that individuals can be inaccurate and may alter dietary patterns when asked to report their dietary intake [27,28]. In our experience, Italian males with fertility problems usually report almost full adherence to a Mediterranean diet even when their BMI index and clinical parameters strongly suggest unhealthy diet habits [unpublished data]. This represents a real weakness in any kind of diet strategy based on reducing the intake of dangerous food since patients often are not fully aware of their diet habits.

In the last decade about >2 million direct-to-consumer tests have been sold by different companies. These tests can be ordered on the internet without any medical prescription and are often offered under the label of “lifestyle” genetic testing, by-passing the more stringent legislation covering clinical and medical devices/services [29,30]. Ethical and legal issues are involved in these procedures, including the complexity of data interpretation, doubts about the clinical significance of results and management of the information. Finally, establishing for whom and to what extent a nutrigenetic test is considered clinically useful can be controversial. In addition to the above, the confusion generated about the meaning and scope of their potential results for consumer/patient has been seriously questioned, as such tests can generate unrealistic hopes or cause a false sense of security or undue anxiety [30,31]. Although this debate is still ongoing, we underline that both pre-test and post-test genetic counselling should be provided for nutrigenetic panels. Several elements should be addressed before and after nutrigenetic testing including: (i) the nature of the test and its results, (ii) utility of the test, (iii) meaning/scope of the results, and (iv) risks [30].

However, another important topic in this field is the recent discovery that not only can a specific genetic variant influence the metabolism of specific foods, but diet itself is able to modify gene expression. This leads us to discuss one of the most interesting issues in this field, that is, the epigenetic consequences of diet on human reproduction.

## 4. Epigenetics, Diet and Human Health

Epigenetics focus on the molecular processes that modulate gene expression without changing the DNA sequence, such as DNA methylation, histone modification, and microRNA (miRNA) regulation [32]. In this context, to date, nutritional epigenetics, that is, the study of changes in gene expression induced by bioactive dietary compounds, is emerging as a novel topic in studies investigating the impact of nutrition on health [33] (Figure 1).

Nutrition is one of the most modifiable factors able to affect DNA methylation pathways. It has been demonstrated that nutrition can influence the epigenetic regulation of DNA methylation in different ways by altering the substrates and cofactors necessary for this process, by changing the activity of enzymes regulating the one-carbon cycle or by playing a role in DNA demethylation activity [34].

The most important effect of diet on the epigenetic modulation of gene expression is represented by early life nutritional experiences that are able to induce persistent metabolic and physiological changes through altered epigenetic profiles, leading to different susceptibility to various chronic diseases in later life [35]. In this view, prenatal exposure to different elements plays a critical role.

In fact, maternal malnutrition and/or over-nutrition during the pre and postnatal period are the main stressors that are able to influence offspring outcomes and adult phenotypic consequences, by increasing the susceptibility to metabolic disease [2,36].

An epigenetic link between unbalanced maternal diet during pregnancy, fetal growth and CVD risk in adulthood has been consistently demonstrated, for example, DNA methylation levels at genes regulating cortisol levels, tissue glucocorticoid action and blood pressure have been associated with both early-life parameters and cardiometabolic risk factors [37]. Significant seasonal variations in maternal methyl-donor nutrient intake during the periconceptional period influence several maternal plasma biomarkers that predict changes in the methylation at metastable epialleles in lymphocytes and hair follicles in infants postnatally [38]. In addition, maternal obesity can lead to DNA methylation changes, which are present at birth and remain postnatally. In fact, the study of maternal obesity, with or without GDM, has shown many differentially methylated sites in DNA from the umbilical cord blood of offspring, and from 4–5 year-olds and 9–16 year-olds [39,40].

Thus, maternal nutrition can be considered as a major influence on resetting the epigenome in the early embryo because it affects offspring phenotype through alterations of oocyte maturation, oocyte provisioning, and oocyte stores of mitochondria and metabolites. In particular, the cytoplasmic constituents respond to maternal nutrition in a specific way: dietary fat increases lipid droplet size and composition, micronutrients influence DNA methylation and alterations in dietary lipid and sugars affect mitochondrial activity [41]. In this regard, the oocyte quality, mitochondrial function, and fertility in animal models can be restored by caloric restriction or omega-3-enriched diet, which suggests that these alterations can potentially be modified by nutritional or targeted therapeutic interventions [42,43]. In addition, it has been demonstrated that maternal methyl donor supplementation can reverse DNA hypomethylation induced by endocrine-disrupting chemicals in early development [44].

Recent studies have confirmed that males also transmit epigenetic modifications to the offspring, thus influencing not only embryo growth but also lifetime health [44,45].

Epigenetic alterations can affect male germ cell development at different phases. Environmental or lifestyle insults such as toxins, endocrine disrupters, smoking, and obesity can affect sperm during development in the testes or during maturation in the epididymis [46,47]. In particular, it has been demonstrated that sperm from obese glucose-intolerant males show distinct small noncoding RNA (sncRNA) expression and DNA methylation profiles as compared to lean, normal-glucose tolerant subjects. Alterations in DNA methylation affect differential methylation clusters within genes known to contain SNPs related to obesity, such as *FTO*, *MC4R* and others. In addition, remodeling of the obesity-associated sperm DNA methylation pattern in a separate cohort of men as a result of bariatric surgery has also been observed. This specific remodeling involved gene regulators of appetite control (such as *MC4R*, *BDNF*, *NPY*, *CR1*) or metabolism (such as *FTO*, *CHST8*, *SH2B1*) [48]. These results were corroborated by Soubry et al. [49] who confirmed that male overweight and obesity status is traceable in the sperm epigenome. In fact, these authors demonstrated lower methylation percentages at the *MEG3*, *NDN*, *SNRPN* and *SGCE/PEG10* differentially methylated regions (DMRs) as well as a slight increase in DNA methylation at the *MEG3-IG* DMR and *H19* DMR in sperm of overweight or obese men.

Although the above reported studies do not make it possible to clarify if altered sperm methylation was due to obesity itself or to the dietary lifestyle of the patients, taken together these observations demonstrate the presence of a link between nutrition and sperm epigenetic patterns. Therefore, it is possible that sperm epigenetic modifications can be transmitted to the offspring, which may lead to paternal epigenetic inheritance of metabolic disorders (paternal origins of health and diseases (POHaD)) [50]. Thus, it has been proposed that a nutrition-linked mechanism passed through the male line is able to influence the longevity and the risk for cardiovascular and diabetes mellitus mortality when either the father or the paternal grandfather have been exposed to an excess of food from 9–12 years of age [51,52,53].

Thus, the modifications of the epigenetic landscape by dietary compounds can affect overall health but also the reproductive health of both sexes, and the stressors of both parents, even before conception, and they can shape the development and life-course trajectory of the embryo and fetus [47,54,55,56].

In light of the above, the “epigenetic diet” could be a promising approach to neutralize epigenomic aberrations caused by exposure to environmental contaminants. Increasing evidence has shown the beneficial health outcomes induced by bioactive dietary compounds such as isothiocyanates in broccoli, genistein in soybean, epigallocatechin-3-gallate in green tea, resveratrol in grape, and ascorbic acid in fruits, which modify the epigenome [57].

## 5. Other Viewpoints

Although current evidence is accumulating to support the interplay between parental diet, genes and offspring health, conversely, some works identify the failure of the gene-centric (i.e., DNA and epigenetic) paradigm in clinically relevant research [58].

Following an analysis of the history of the gene-centrism perspective and reassessing the fundamentals of evolutionary theory, Bonduriansky et al. suggested that non-genetic inheritance, which encompasses epigenetic, environmental, behavioural, and cultural factors, could play an important role in evolution, by imposing transgenerational effects and generating heritable variations in a broad array of traits in all organisms [59,60,61].

The maternal resources hypothesis proposes a novel conceptualization of inheritance and evolution, in which non-genetic vectors, including accumulative maternal effects (i.e., maternal prenatal energy metabolism and maternal postnatal physical activity), socio-environmental and phenotypic evolution, are the predominant causal factors for the health of future generations [62]. Thus, in this evolutionary context, the “overconsumption” that leads to metabolic disease is due not to dietary factors per se, but rather to physical inactivity caused by increments in energy intake and non-genetic evolutionary processes with the adipogenic partitioning of nutrient-energy [62,63,64].

In this critical hypothesis, diet is an essential component of health, however it may be a trivial risk factor in the case of chronic diseases [65,66], suggesting that macronutrients can have metabolic effects dependent of the individual physiologic context (e.g., physical activity level) [65]. In addition, the role of diet in chronic diseases has been highly controversial [66]. Some of this controversy was prompted by the publication of epidemiologic reports supporting memory-based methods to measure dietary intake [27,28,65,67] even though these methods can produce inaccurate dietary data collection, resulting in spurious associations and effects [27].

Considering these discordant viewpoints, further interventional and longitudinal research with a rigorous approach is essential in order to explore the possible role of epigenetic mechanisms and diet as mediators of the consequences for future generations.

## 6. Future Perspectives

There are great expectations for nutrigenetics, especially in regard to studies related to its future beneficial application in health promotion and for personalized nutrition in the prevention of chronic illnesses. Although studies that provide a comprehensive understanding of the interaction between diet, genes and human health require further effort, the identification of optimal interventions could modulate pre-existing genetic risks, thus reducing the susceptibility to lifestyle-related diseases. To that aim, the notions of nutrigenetics and nutrigenomics should no longer be considered as two independent mechanisms of correlation between food and gene expression, but should rather be seen as two interacting patterns.

Recently, it has been suggested that lifestyle modifications, including personalized diet and physical activity intervention, may impact on obesity through changes in the expression level of the *FTO* and *IRX3* genes. However, this effect is mediated by the genotype of *FTO*, which is capable of modulating the impact of lifestyle changes on its own expression [68]. These findings have highlighted how important gene variants related to nutrients and metabolism are, especially in terms of a possible link between dietary intake and gene expression and their related functions [69].

On the other hand, attention must also be paid to nutrigenomic studies that focus on the role of nutrition in the health of individuals and their offspring.

Over the last three decades, some evidence from both animal and human studies has indicated epigenetic mechanisms as possible mediators for developmental programming of obesity and T2DM via parental exposure. In general, studies have focused on one of three broad environmental factors, both during and before pregnancy: (i) altered diet/nutrition, (ii) toxin exposure, and (iii) stress and have demonstrated alterations in the course of embryonic development and possible phenotypic changes into adulthood. As for the diet, animal model research has identified tissue-level epigenetic alterations in the fat, muscle, pancreas and liver biopsies from the offspring of mothers fed with specific diets [70,71,72,73,74]. On the other hand, due to ethical and clinical limitations, most human studies have examined epigenetic profiles mainly in placenta, offspring umbilical cord or infant blood as surrogate markers of metabolic tissue-level epigenetic modifications [39,40,75,76,77,78,79]. The vast majority of these studies have focused on DNA methylation, while miRNA expression and histone modifications need to be better understood. In this regard, miRNAs have been investigated as possible biomarkers of epigenetic modification in maternal diet-induced obesity, especially in mice. Maternal high-fat diet and a high simple-carbohydrate diet may cause a programmed increase in *miR-126*, leading to a reduction in the insulin receptor substrate-1 expression in epididymal white adipose tissue of male offspring [80]. Moreover, maternal high-fat diet consumption can affect the early lipid metabolism of offspring by modulating hepatic β-oxidation-related genes and miRNA expression [81]. Taken together, these findings strongly suggest that such mechanisms may contribute to metabolic disturbances in adult life.

Although the literature is mainly concerned with maternal epigenetic and gestational effects, there is evidence that supports paternal contributions in modulating an offspring’s health outcomes [81,82,83,84,85,86]. The findings from animal studies show clearly that the dietary perturbation (e.g., high-fat diet) can influence several phenotypes in the next generation including body weight, fat distribution, abnormalities in glucose tolerance, as well as reproductive health [83,87,88,89].

On the other hand, undernutrition, such as low protein diet, caloric restriction or intermittent fasting, can also have an impact on progeny phenotypes including changes in cholesterol and lipid metabolism, glucose control, and other cardiovascular risk factors in the offspring [85,90,91,92,93,94]. In fathers who were fed a low-protein and high-fat diet, sperm cells displayed global DNA hypomethylation and altered miRNA expression [89].

In addition, epigenetic stressors including endocrine-disrupting chemicals, alcohol and nicotine abuse, can cause intergenerational reproductive health and metabolism effects [95,96,97,98,99,100]. In the context of endocrine-disrupting chemicals, transgenerational effects on brain, behavior, and reproduction have been documented [95,101,102,103]. For example, in one of the earliest studies, which reported the paternal effect, it was demonstrated that the exposure of pregnant rats to high levels of vinclozolin during fetal gonadal development induced decreased sperm number and motility in F1, F2, F3, and F4 generations, with 8% of males developing infertility [95]. Recently, some studies have supported the idea that various RNAs in testis, spermatozoa, and seminal fluid can act as epigenetic vectors of inheritance by which paternal environmental state influences metabolic and non-metabolic phenotypes in offspring [104,105,106,107]. Particularly, miRNAs in testis may modulate spermatogenesis, while miRNAs in spermatozoa and seminal fluid may influence early fetal development through interactions with the endometrial environment [50]. Although it is increasingly clear from animal models that the sperm epigenome carries some information from father to child, this evidence is still lacking in humans and it should be better explored.

Although the current evidence has been questioned by some studies [58,64,108], the data in the literature are accumulating in support of the potential role of epigenetic and genetic patterns of parents in offspring health. Therefore, comprehensive knowledge of the epigenetic modifications induced by unhealthy parental lifestyles or by exposure to environmental insults in the periconceptional period could provide useful insights for the prevention of long-term disease in the offspring. There is emerging data that suggest the predictive power of epigenetic markers in gametes. For example, the studies of Jenkins et al. [109] demonstrated the utility of epigenetic screening of mature sperm for the identification of various fertility-related diseases and for successful assessment in assisted reproductive techniques.

Dietary patterns, nutrients and bioactive compounds interact with metabolic traits through epigenetic mechanisms; thus, they represent attractive therapeutic targets. Recently, a potential protective role of bioactive dietary compounds in neutralizing epigenetic aberrations induced by several environmental factors has been suggested. [57]. Nutrigenetics, nutrigenomics, as well as epigenetic diet are being more widely explored so that their implementation can become an innovative and effective measure for the protection of human health, especially for future generations.
